# Tortures alleged by migrants in Italy: compatibility and other medicolegal challenges

**DOI:** 10.1007/s00414-021-02646-4

**Published:** 2021-07-06

**Authors:** Ilenia Bianchi, Martina Focardi, Valentina Bugelli, Francesco Pradella, Carlo Giolli, Francesca Friani, Vilma Pinchi

**Affiliations:** 1 Forensic Medical Sciences, Health Sciences Department, University of Florence, Largo Brambilla 3, 50134 Florence, Italy; 2 Medico-Legal Service of National Health System in Florence, Florence, Italy

**Keywords:** Torture, Ill-treatments, Asylum seeker, Medicolegal report, Istanbul protocol, Abuse, Irregular migrant

## Abstract

**Background:**

Illegal immigration to Europe is a well-known phenomenon whose numbers are being steadily increasing in recent years. Most of the immigrants in Italy come from war zones, and many of them submit an asylum application supported by the complaint to have been victims of persecutory acts in their home countries.

**Material and methods:**

One hundred ninety-six medicolegal reports are analyzed considering the different country of origin, the type of the lesions claimed, tools used, evidenced effects, location of the perpetration of the physical abuses, and the possible motivation of the alleged torture.

**Results:**

Greater than 80% of the assessed asylum seekers are over 18-year males coming from African countries. Fifty-eight percent of migrants were tortured or abused in countries of transit, 95% in Libya. Economic, familial, politic, and ethnic reasons prevail in some countries of origin, while tortures or abuses perpetrated in transit countries are mainly linked to forced labor and detention. In the 42.2% of cases, no physical evidence of tortures was detected. The Istanbul Protocol resulted to have been only partly applicable and about 40% of the medicolegal reports are “inconclusive” about the compatibility of physical evidence with the alleged tortures.

**Conclusions:**

The medicolegal and forensic experts involved in torture and ill-treatment cases should seek specific education and training to lower the risks of underestimation and the rate of inconclusive reports. More extensive implementation of the Istanbul Protocol in daily practice should be pursued by the authorities in charge of asylum or protection releasement.

## Introduction

Illegal immigration to Europe is a well-known phenomenon whose numbers are being steadily increasing in recent years. According to the United Nations High Commissioner for Refugees (UNCHR), the number of forced immigrants reached a total of 70.8 m in the world in 2018, 25.9 m of whom with the refugee status and 3.5 m still waiting for the outcome of their application as asylum seekers.

Most of the immigrants in Italy come from war zones and many of them submit an asylum application supported by the complaint to have been victims of persecutory acts in their home countries [1]. Forensic assessments are often required by the local Committees in charge of granting or refusing the international protection status which implies political asylum, or, if the requirements for the “refugee status” are not fulfilled, the subsidiary protection and special temporary permits.

International and national guidelines [2, 3] have been issued for the recognition and inclusion of the immigrants who can show a “well-founded fear to be subjected to a personal prosecution in their home country according to the Geneva Convention.”

The forensic physicians are charged with cases of illegal immigration for two main important purposes: age estimation in unaccompanied minors and forensic assessment of alleged torture acts according to the Istanbul Protocol (IP) [2].

To follow and enact at best the suggestions of the IP, a dedicated forensic service for the reporting and verification of torture or physical abuse cases claimed by asylum seekers was activated at the ASL Toscana Centro in Florence in 2014.

The literature specifically dealing with physical lesions in individuals claiming to have been victims of tortures or ill-treatments is rather scarce, and therefore, it could be difficult for a European physician to acquire a specific knowledge or experience in such a peculiar matter as this area of forensic medicine is. From the analysis of the recent literature [4, 5], it is easy to realize that the physicians involved in the assessment of torture or abuse acts seek appropriate training to improve their knowledge of some specific lesions.

The present study analyzes a sample of forensic reports about asylum seekers who claim to be victims of tortures in their home countries or during the migration journey. The research emphasizes the type of physical injuries suffered by the asylum seeker victims of torture and the judgement of consistency expressed in the forensic assessments required by the competent authorities. The potentiality and limitations of the assessments of torture cases following the IP recommendations are discussed.

## Materials and methods

The authors analyzed the forensic reports issued by the dedicated service of the ASL Firenze and required by the local Committee for the Asylum Seekers in the 2014–2018 period of time.

The reports have been anonymized by the forensic service officers and their sections examined as follows:

- anamnestic interview, in which the asylum seeker talks about modality, causes, and when and where he/she suffered from the alleged physical abuses and describes the symptoms
- external examination
- possible further examinations/investigations (specialistic, radiographic, other examinations)
  A descriptive analysis of the sample has been made. Three main groups of variables have been considered and clustered:
  - Demographic data of the asylum seekers: age, gender, country of origin, and transit countries.
  - Type of the lesions claimed and tools used, evidenced effects, country of perpetration of the physical abuses (home or transited countries), and the possible motivation of the alleged torture acts. The lesions have been classified in three macro-categories according to the IP depending on their compatibility in causing the physical effects evidenced at the external examination [2]:
    - No physical lesion observed
    - Nonspecific lesions;
    - Specific lesions.
    - These sub-categories have been identified according to the classification of the IP^2^.
  - Applied methodology according to the IP: the medicolegal judgement about the degree of compatibility between the declared story of physical violence and alleged torture acts and the data obtained from the external examination.

The epidemiological data have been then examined with a special focus on the victim’s history and the possible unrelated accidental impairments declared by the applicant himself.

## Results

One hundred ninety-six reports issued by 4 forensic physicians (3 males and 1 female), always working in pairs, have been collected. Of the whole sample, aged between 16 and 46, 194 reports were about male individuals and 2 about females.

The demographic characteristics and the country of origin of the individuals are displayed in Table 1.

**Table 1:** Demographic data of the asylum seekers

Gender	Male	194
	Female	2
	Tot	196
Age	Min.–max	16–46
	Average age	25.3
	Age range (%)	
	< 18	17 (8.6%)
	18–21	30 (15.3%)
	22–40	138 (70.4%)
	> 40	11 (5.6%)
Country of origin*	West Africa tot. (%)	160 (82%)
	Nigeria	50
	Guinea-Conakry	22
	Gambia	22
	Senegal	19
	Mali	18
	Ivory Coast	17
	Ghana	6
	Guinea-Bissau	2
	Liberia	2
	Burkina Faso	1
	Sierra Leone	1
	East Africa tot. (%)	5 (2.5%)
	Somali	3
	Eritrea	2
	South-Central tot. (%)	26 (13%)
	Pakistan	15
	Bangladesh	9
	Afghanistan	2
	Europe tot. (%)	3 (1.5%)
	Middle East tot. (%)	2 (1%)
Transit country*	Tot. (%)	112 (57%)
	Libya	106
	Bulgaria	2
	Burkina Faso	1
	Dubai	1
	Gambia	1
	Turkey	1

^*^84 subjects (43%) declared to have been submitted to maltreatments in the home and transit countries

Most of the individuals are in the 22–40-year group (70.4%) while 17 individuals (8.6%) declared to be under 18. The most represented origin area is West Africa (160 individuals, or 82%), and 83 subjects (42%) claimed tortures perpetrated in the country of origin, 29 subjects (15%) only in the transit countries, and 84 subjects (43%) declared to have been submitted to abuses both in the origin and in transit countries. Overall, 58% of migrants were tortured or abused in the countries of transit, and the 95% of them (106/112) in Libya. In one case only, a minor was submitted to an age estimation procedure.

The presence of a psychosocial and linguistic mediator during the visit was always assured whenever problems of comprehension had been signaled. The mental health local services were charged with the psychiatric problems; this aspect is not considered in the present research.

Table 2 shows the type of alleged lesions related to the incidence in the home country and the country of transit (panel B) and the motivation for the alleged torture acts (panel A). Physical violence perpetrated in African countries finds a motivation in familial and political, followed by religious, ethnic, and economic reasons, which resulted indeed prevalent for migrants from Bangladesh and Pakistan. Only a few migrants coming from Africa reported to have been reduced in slavery or sexually discriminated against. Subjects in transit through Libya reported physical violence acts when asked to pay the fee to the smugglers or the border police. The lesions resulted to be caused by detention in inhuman conditions and forced labor. Sometimes the same subject (especially those who suffered abuses also in the country of transit) declared to have been submitted to differently motivated multiple acts of violence. Specific injuries inflicted with rifles or military boots or foot whipping (the so-called falanga), scourging (using typical multi-thong ropes ending with a set of metal balls or bones or metal spikes, similar to “roman flagrum” or flail), or electrocution were mainly reported by migrants who crossed Libya. Lesions caused by acts such as whipping (using nonspecific whips, rods, ropes, or cables) or slashing (using sharp weapons, e.g., ax or machete) or burning are allegedly inflicted in African countries, being cigarette burns prevalent in Libya-related cases. Forced avulsion of teeth or nails is claimed by migrants coming from Mali. Death threats were mostly reported by those who escaped from Africa, Bangladesh, and Pakistan. Violent interrogations by the police in order to obtain information and confessions were reported by some subjects from Bangladesh and Pakistan. From the analyzed medicolegal reports, it emerges that 638 overall alleged injuries related to torture acts or ill-treatments were reported to the forensic physicians.

**Table 2:** Motivations and methods of execution of the alleged tortures related to the main home and transit countries

Panel A—reasons for alleged torture	Africa	n (%)	Bangladesh and Pakistan	n (%)	Libya	n (%)
Economic	14	63.6%	7	31%		
Political/police	28	70%	9	23%		
Familiar	38	88.3%	4	9.3%		
Religious	17	89.4%	2	10.5%		
Ethnic	19	95%	1	5%		
Slavery	8	100%				
Forced recruitment in civil war *(young soldier)*	2	66.6%	1	33.3%		
Sexual discrimination	8	100%				
Payment of fees (smugglers/police)					106	96%
Panel B—injuries referred as alleged consequence of torture	Africa	n (%)	Bangladesh and Pakistan	n (%)	Libya	n (%)
No scars detectable						
Detention					93	100%
Forced labor	7	19.4%			33	82.5%
Food deprivation	6	43%			8	57%
Poor hygienical conditions in detention					6	100%
Medical treatment deprivation	3	33.3%			6	67%
Sexual abuse	4	50%			4	50%
Light deprivation	1	25%			3	75%
Butcher-like suspension	1	50%			1	50%
Death threats	9	64%	5	36%		
Violent interrogation			3	100%		
Generic injuries						
Assault with pointed instruments	8	61.5%	1	7%	4	30%
Blunt instrument beating	77	56%	21	15%	39	28.4%
Assault with sharp instruments	75	73%	13	12.6%	15	14.5%
Specific injuries						
Rifle butt assault	2	16.6%			10	83.3%
Assault with sharp instruments used to slash	3	100%				
Restraint	11	60%	1	6.6%	5	33.3%
Falanga (foot whipping)					9	100%
Gunshots to stop escape	7	50%	1	7%	6	42.8%
Military boots assault					3	100%
Scourging (*flagrum/flail*)					2	100%
Whipping (nonspecific flexible instruments)	11	73.3%			4	26.6%
Amputation following an explosion	1	50%			1	50%
Nail extirpation	2	100%				
Teeth avulsion	1	100%				
Nonspecific red-hot instrument or boiling liquid burn	24	80%			6	20%
Cigarette burn			1	33.3%	2	66.6%
Electrocution			2	16.6%	10	83.3%

Table 3 displays the different kinds of tortures and the related clinical findings that ranged from the condition of “no lesion” to those of “nonspecific clinical lesions” and “specific clinical lesions” according to the subject’s reporting during the interview, registered during the clinical examination. On 638 referred lesions, 203 were ascribed to beating, sometimes performed with a blunt instrument, but 21.1% of them left no visible after-effect signs. In the other cases, 50.7% left nonspecific scars while 15.7% caused a painful limitation or functional impairment, and 12.3% radiograph-detectable signs of fracture, or osseous deformities, loss of teeth, or dental prosthetics following beating to the face (about 10 cases).

**Table 3:** Methods of execution of the alleged torture-related to the external physical signs

Kind of tortures or ill-treatments	No lesions, n (%)	Nonspecific clinical lesions	n (%)	Specific clinical lesions	n (%)
Waterboarding	1 (100%)				
Medical treatment deprivation	9 (100%)				
Forced recruitment in civil war (*young soldier*)	3 (100%)				
Death threats	14 (100%)				
Detention	93 (100%)				
Food deprivation	14 (100%)				
Light deprivation	4 (100%)				
School education deprivation	2 (100%)				
Poor hygienical conditions in detention	6 (100%)				
Forced labor	40 (100%)				
Violent interrogation	3 (100%)				
Slavery	8 (100%)				
Butcher-like suspension	1 (50%)			Axilla and/or upper hip cuts	1 (50%)
Blunt instrument beating					
Generic beating/nonspecific blunt instrument	43 (21.1%)	Nonspecific scars	103 (50.7%)	Fractures PDF NDF PDFOD TLTDP	25 (12.3%) 2 (8%) 4 (16%) 9 (36%) 10 (49%)
				PLFI	32 (15.7%)
Restraint	9 (53%)			SSHCTW on ankles and wrists	8 (47%)
Falanga	4 (44.5%)			SCWFC	5 (55.5%)
Whipping	1 (6.6%)			PSLCW	14 (93.3%)
Scourging				FTS	2 (100%)
Sexual abuse/rape	6 (75%)			SLCW in internal thigh	2 (25%)
Assault with sharp instruments		Nonspecific scars	93 (91.1%)	SSDW on wrist forearm hand back	9(8.9%)
Assault with pointed/penetrating instruments		Nonspecific scars	13 (48.1%)	Gun scars “Entry hole” “Entry-exit hole” “Graze wound scar”	14 (51.8%) 5 (35.7%) 5 (35.7%) 4 (28.5%)
Assault with sharp instruments used to slash				Deep scars	3 (100%)
Burns					
Red-hot instrument or boiling liquid burn				Burn scars Extensive (e.g., hot water, acid, plastic…) Localized CTS	32 (100%) 9 (27%) 20 (60%) 3 (9%)
Electrocution	8 (66.6%)			EMS	4 (33.4%)
Amputation					
		TCWAB	2 (100%)	HFNE	3 (100%)
		BIF	3 (100%)	DPLB	1 (100%)
Total	269/638 (42.2%)		214/638 (33.5%)		155/638 (24.3%)

*PDF*, positive diagnosis of fractures after instrumental exam; *NDF*, negative diagnosis of fractures after instrumental exam; *PDFOD*, positive diagnosis of fractures due to the presence of osseous deformities without radiographs; *TLTDP*, traumatic loss of teeth or dental prosthesis; *PLFI*, painful limitation and/or functional impairment; *SSHCTW*, symmetrical scars and hyperkeratosis due to compression/traction wounds; *SCWFC*, scars due to contused wounds and foot sole callosity; *PSLCW*, parallel scars caused by lacerated-contused wounds; *FTS*, flagrum typical scars; *SLCW*, scars caused by lacerated-contused wounds; *SSDW*, scars related to self-defense wounds; *EMS*, electric mark dark margin round scar; *CTS*, circular tattoo scars related to cigarette burn; *TCWAB*, terror or civil war acts bombing; *BIF*, burn after intentional fire; *HFNE*, hand or foot nail extirpation; *DPLB*, dental torture, tooth loss, prosthesis loss after beating

The injuries reported as due to sharp and pointed/penetrating instruments caused mainly nonspecific scars. Specific defensive injuries on the upper limb have been found in 9% of cases, and in about 70% of the gunshot wounds, the typical signs of the entry hole were found. Generally speaking, 42.2% of the examined cases did not show any physical evidence of torture. Nonspecific or specific injuries were found in 33.5% and 24.3% of cases, respectively.

Concerning the type of alleged torture generally causing more specific injuries, we found that 9% of burning lesions had the typical cigarette burn morphology while the rest showed localized red-hot instrument injuries or acid/hot water extensive lesions. Electrocution was deemed responsible for typical scars only in 33% of cases; physical signs of sexual abuse were found in 25% of cases; falanga (foot whipping), body restraint, or suspension caused permanent scars in 50% of cases; whipping in the 90% of the subjects; and nails or teeth avulsion, scourging, and slashing injuries in 100% of cases. Acts of torture such as forced labor, deprivation, detention, and death threats typically do not leave any detectable signs.

Radiographs were available in 3% of cases only, and no further radiographs or instrumental examinations were required by the medicolegal specialists.

Table 4 shows the results of the medicolegal assessments. Firstly, it shows that the physicians did not strictly follow the Istanbul Protocol, which comprises 5 steps of compatibility between the lesions and the alleged tortures, ranging from “not consistent” to “consistent,” “highly consistent,” “typical,” and “specific.” In the examined sample, however, only the following compatibility classifications were adopted: 1—“incompatible”; 2—“compatible”; 3—“inconclusive.”

**Table 4:** Degree of consistency between the overall pattern of lesions and the torture alleged by the asylum seekers (196 cases)

Compatible		Incompatible		Inconclusive			
				Only the individual lesions were described		Only some injuries were deemed compatible with tortures	
	N (%)		N (%)		N (%)	Incompatible Lesions	N (%)
Nonspecific scars, fractures, burn or electrocution scar, painful limitation and/or functional impairment, whipping, scourging, gunshot, slashing, amputations	114	Contusive trauma after kicks and beating not related to preexisting pathological condition	2	Nonspecific scars, fractures, burn and electrocution scars, painful limitation and/or functional impairment, whipping, scourging, gunshot, slashing, amputation	48	Preexisting pathologies (e.g., nontraumatic back pain, arthritis, preexisting stuttering, petechiae, hiatal hernia, and infection	6
		Maltreatment without detectable visual or radiograph effects	1	Dental torture (with bricks and pliers)	1	Electrocution	3
		Falanga without detectable effects	1	Incompatibility between the alleged instrument and detected scars (smear gunshot, unusual cutting scar, unusual burn, and electrocution scar)	3	Accidental fractures voluntarily declared	2
		Foot nails mycosis	1	Amputations and/or blast scars	2	Falanga	1
				Nonspecific injury without further detectable effects related to torture (e.g., generic back and face beating with teeth loss, sexual abuse, whipping)	9		
				Nonspecific injuries related to road accidents voluntarily declared	2		
Total	114 (58.1%)		5 (2.5%)		65 (33.2%)		12 (6.2%)

The whole injury pattern was declared consistent with the claimed torture act in 114 (58.1%) of the 196 examined cases, and only in 5 cases (2.5%), the medicolegal expert issued a report of exclusion. In 77 cases (39.2%), the reports expressed an inconclusive judgement: in some of them (33.1%), only the individual lesions were described, while in other 12 cases (6.1%), a not homogenous pattern of injury—only partly ascribed to torture—was described. In four cases, the asylum seeker autonomously declared the accidental origin of the injuries which were not to be included in the torture lesion pattern.

In 3% of cases, a preexisting specialistic examination (gastroenterological, neurological, orthopedic) led to the exclusion of injuries related to congenital malformation (hip, testicle, etc.) rather than to violent acts. No further specialistic or instrumental examination was required.

## Discussion

The asylum seekers who allegedly suffered torture acts or ill-treatments should be submitted to a forensic examination for the assessment of the presence of torture-related psychophysical injuries according to the Istanbul Protocol [2].

The literature based on the forensic assessments of subjects who allegedly suffered torture acts is rather scarce [6–20]. It is therefore so difficult for the medicolegal or forensic specialists to acquire a specific knowledge and experience in the field that the matter of the evaluation of the physical signs of torture is still considered a challenging area of forensic medicine [21]. The physician involved in this specific area might not, therefore, be expert enough to detect some peculiar injuries related to specific kinds of torture acts. We should consider, however, that despite the increasing number of asylum seekers in Italy during the 2014–2017 period of time, a low percentage of the total number of the submitted applications has been effectively examined [22]. This fact can overshadow the real magnitude of the phenomenon and the real number and type of tortures or ill-treatments claimed by the asylum seekers [11, 13].

The large prevalence of male subjects in the examined sample (Table 1—99% males) is consistent with previous reports [9–17]. In a 2017 UNHCR report, the number of female subjects represented only 11.2% of the migrants across the Mediterranean Sea, with a prevalence of Nigerian and Eritrean women [23]. Mainly economic and familiar reasons discourage women to migrate, but other factors could explain the very low number of women included in the sample examined here. According to the International Organization for Migration (IOM), about 80% of Nigerian women who migrated to Italy in 2016 have been smuggled for sexual exploitation; therefore, such victims, living in an illegal status, are very seldom prone to report to have been subjected to violence or torture [9–17]. Moreover, the local Committee for asylum anecdotally reports that the asylum-seeking women frequently obtain a positive response for reasons related to children, pregnancy, abusive husbands, etc. other than torture acts. By doing so, the real number of torture acts perpetrated against women is blacked and the assessment of the real distribution for sex of the victims of torture is in the same way jeopardized. On the other hand, the large prevalence of sub-Saharan West African subjects in the examined sample is consistent with the data of 2016 AFIC/FRONTEX report [24]. About 89% of the migrants arriving in Italy in 2016 came from Africa and the asylum seekers from sub-Saharan West Africa represented more than 50% of sea arrivals. Nigeria was the most represented nation, from where came 14% of immigrants arrived in Europe. West Africa is the area where largely prevailed the recruitment, transit, and exploitation for human trafficking animated by local and international organizations [24]. According to a 2015 IOM report, the majority of arrivals to Italy came from Eritrea, Nigeria, Somalia, Syria, Gambia, and Sudan [25]. Our data are only partially in accordance with the Italian data reported in previous studies. Franceschetti et al. [11] found that 85% of asylum seekers in Milan came from African countries, while Di Napoli el al. [12] reported that 51% of asylum seekers originated from Asian countries; thereby, it is impossible to compare the immigrants’ and asylum seekers’ origin at national level.

Asylum requests from minors (< 18) or young adults (18–21) were 8.6% and 10.6%, respectively (Table 2). The coming of age is reached at 18, but according to the Italian laws, the protection can be extended up to 21 if the existence of social difficulties is established by a Minor Court. The number of minors is very limited in our sample, as it is in previous studies and as was largely expected since children receive anyway protections just for being minor regardless of the possible presence of torture injuries or ill-treatments. This fact may conceal the real occurrence of torture injuries among children or young adult migrants, thus limiting the full appreciation of the phenomenon and the consequent treatment of the physical/psychological lesions.

Most of the subjects examined in this study started their migration journey in sub-Saharan Africa, Bangladesh, and Pakistan, and this data are consistent with Amnesty International’s annual report about the difficult political, civil, and discriminatory situation in these geographic areas. In our sample, about 85% of the subjects claimed to have been tortured in the country of origin (43% only in the country of origin; 43% in both the origin and transit countries) (Table 2), while the 15% claimed to be abused only in a transit country. The latter data is pretty relevant from a legal point of view, considering that the asylum is granted only to the victims of torture in the country of origin, while this protection is refused if the torture is perpetrated during an illegal migration and in transit countries (e.g., in Libya).

The analysis of the sample characteristics showed no real correlations between the type of the alleged tortures and the country of origin or transit. A relevant prevalence of the causes of the torture acts emerged from the analyzed sample, being the familiar contrasts or political fights the prevalent causes in African countries and political and economic reasons in Bangladesh and Pakistan. Libya was the main country of transit, and the tortures perpetrated there were linked to labor exploitation for the payment of the smuggling fees or to physically weaken the subject or as a punishment for trying to escape the camps. According to the results of the paper by Reques and coll. [26], the Central Mediterranean Route, across Libya, is one of the most dangerous but otherwise the preferred one by the West African migrants. About 50% of the male migrants across Libya are detained and forced to work until they are able to pay the ransom, for an average time of 3–4 months.

The analysis of the causes of the different kind of tortures reveals that the asylum seekers who claimed to have been subjected to violence in Libya reported mainly physical ill-treatments and sufferings perpetrated with deprivation techniques (food, water, sensorial deprivation, medical treatment refusal) during detention or forced labor, or more typical kinds of injuries such as electrocution, cigarette burns, beatings with military equipment tools.

Tortures perpetrated for political reasons range from generic physical violence (beatings with or without blunt instruments) to deprivation techniques (food, water, sensorial deprivation, medical treatment refusal) but also to more specific injuries (restraint, whipping, etc.).

According to the more recent literature [9, 11], few subjects describe torture acts with guns which are instead sometimes used in beatings as a blunt instrument.

The tortures perpetrated for religious, economical, ethnic, or familiar (revenge, inheritance) or discriminatory motivations (e.g., ethnic or sexual discrimination) are similar and often consisting in gang assaults with blunt, sharp, or red-hot instruments, ending often with the abandonment of the injured who is not going to receive any help nor treatment for the injuries with consequent extensive and relevant scarring.

Sexual abuse is seldom claimed and is not correlated with gender, torture motivations, or countries of origin or transit. Gender-based and sexual violence is frequently a hidden problem. Although primarily directed at women, in the last decades, there has been a growing reporting of sexual violence against men in particular in the context of political conflict. Forms of sexual tortures described in the previous literature range from verbal sexual harassment, forced nudity, to severe forms of genital violence, such as squeezing the scrotum, rape, genital mutilation, and castration [27, 28]. It seems probable, however, that the real incidence of sexual abuses is largely underestimated in our sample, and that could be ascribed to the low percentage of women in the sample (1%), and to the tendency expressed by the male subjects to hide this type of abuse due to emotions of shame and fear of homosexuality stigma and fear of possible negative retaliation by their own community. Such aspects can explain the low percentage of sexual abuses found in the examined sample in comparison to others in the literature [27].

The study highlights the existence of some specific patterns of injury peculiar or prevailing in subjects coming from specific geographic areas. For example, scourging or electrocution were mainly reported by Libya crossing migrants whereas forced teeth avulsion or nail extirpation are mainly claimed by migrants coming from Mali (Table 2).

The study of the physical injuries claimed by the asylum seeker victims of tortures is especially relevant in our study.

The blunt instrument beating injuries are the most represented in the sample (Table 3), even if they are the most difficult to diagnose with the necessary accuracy [9, 11–13, 15, 18, 19]. There are two main reasons for that they do not always cause detectable signs (21.1%) and the scars are always nonspecific signs because the adopted weapons have inherently nonspecific characteristics (50.7%) [10].

Notably, in the cases in which torture acts with kicks and fists are reported, we did not find any scars, while beatings with the rifle butt or falls on the ground or against hard floors almost always leave scars. In about one-third of cases (28%), we did not find skin scars but the after effect of fractures, painful limitation of movement, or functional impairment [28, 29]. Some blunt instrument injuries can be considered more specific. Whipping, for instance, almost always leaves linear and parallel scars on the back. Scourging with a particular flail/flagrum always causes peculiar signs diagnosed as “typical” injuries by forensic physicians (2 cases).

On the contrary, the claimed “falanga” (a sort of beating on the soles of the feet) and the many types of restraint injuries (parallel and symmetrical scars caused by lacerated-contused injuries on wrists and ankles) have left objective signs in just 50% of cases. In all the other cases, underlying injuries caused by repeated contusions on the soles of the feet or possible tendon and nerve lesions after restraint may be diagnosed only after instrumental or specialistic examination [30], which the subjects in our study have not been submitted to.

In the case of falanga-use, some studies described the importance of imaging exams, such as MRI and scintigraphy [30], which can better define soft tissue alterations and possible fractures.

The traumatic teeth or prosthetics loss caused by blunt instrument beating on the face is a much more frequent type of injury (10 cases) [10] than dental avulsion alone [12, 31], even without any facial bone fracture.

In most cases, the victims of rape did not show any external physical sign of injury even if in 25% of cases a typical scar pattern was detectable on the internal thigh.

Sharp, penetrating, pointed, slashing, and red-hot instrument injuries have left detectable injuries in 100% of cases. Such lesions were defined nearly always as nonspecific, in disagreement with the results of Clement et al. and Ghaleb et al. [9, 10], but in few cases, the injuries were described as specific due to their site and/or morphology and their compatibility with defensive movements (multiple linear cutting scars on the forearm, for instance).

The medicolegal physicians reported difficulties in recognizing scars following gunshot graze wounds.

Second- and third-degree burns always (100%) resulted in a loss of tissue and retracting scars if localized (e.g., red-hot instrument in markings), or extensive scarring (sometimes with a danger of chest tightness during breathing), limitation of joint mobility, or amputations (if caused by acids, hot water, or plastic). The definition of typicity, however, has been granted by the physicians only in 2 cases.

The cigarette circular tattoo on the forearm is a very typical kind of burn which, for its location and morphology, shows a high grade of typicity. The interpretation of such a skin sign is anyway absolutely dependent on the duration and the site of application of the red-hot instrument [9, 18].

A special kind of alleged torture burns has been identified in electrocution cases. The incidence is very low according to most of the previous research [10–12], yet Moisander and Edston [16] reported a high incidence of them in Bangladesh, Syria, and Turkey. Only 25% of cases in our sample had round skin burns with pigmented margins. This type of injury, even if highly characteristic, usually does not cause any detectable sort of impairment [9].

One of the reasons why the attribution of a high level of typicity to most of the injuries has been a difficult issue could be explained by the difficulty of fully understanding the healing process of the different types of skin. This is true, for instance, in the black people’s skin—which represents more than 80% of cases in our sample—which tend to keloid scarring or hypo- or hyper-pigmentation, even in case of normal lesions [18, 28]. The color of the skin can enhance or minimize the effect of scarring and of the detectability of the causative physical violence [18, 19].

The high differential percentage between the number of the examined victims (196) and the detected injuries (638) shows that any asylum seeker is often submitted to many combined types of physical abuses, and therefore, injuries that usually cause undetectable physical scars are often paired to injuries which almost always cause visible scars.

The report analysis of the agreement between the visible signs and the referred history of physical abuse is often complicated for many reasons (Table 4).

First of all, the report results show a methodological lack of homogeneity of the conclusions even though the same four operators performed the visits. The forensic physicians not only did not strictly follow the IP classification/gradation of their judgement of agreement of each and overall pattern of lesions but rather they often (60.7%) expressed only a judgement of “compatibility” or “incompatibility” of the injury pattern with the subject’s referred history.

Furtherly, 39.2% of reports did not consent to draw any valid conclusion, and sometimes, single lesions have been described excluding the presence of only some patterns of injuries (6.1%). This data confirms the difficulty in giving a conclusive judgement about the compatibility of the injuries with the referred torture acts.

In our study, we could not check the true correlation between the medicolegal judgements and the number of protection grants issued by the Committee. Previous research [11, 13] show that the granting of protection is related to the physician’s compatibility judgement which is given mainly following the presence of specific injuries or gun scars, stabbing, and burns (partly), but also following the number and coexistence of different types and modality of the lesions (the more they are and more favorable is the judgement). A retrospective revision is however limiting and highly dependent on the completeness and clarity of the documents provided by the screeners and on the interpretation of the data collected by the controller.

The most important limitation of the study is caused by the fact that the examined reports had not been written for a research purpose and none of them was equipped with photographs [32].

Furthermore, it is not possible to extend the characteristics of the asylum seekers investigated in this study to a national level. In fact, the sample that came to our observation is mainly composed of male subjects aged between 20 and 40 years and coming from sub-Saharan West Africa, for the reasons already discussed. It is therefore impossible to understand what the real incidence of asylum seekers in Italy is in the case of women and minors. It is necessary to consider this type of study as an addition to the contributions to the previous literature.

Moreover, as it has been previously said, in our study, the psychological harmful consequences of abuse on the examined asylum seekers have been excluded since the forensic physician was being charged only with the detection of physical signs of torture. We are absolutely aware, though, that such consequences are undoubtedly the most frequent [16].

The comorbidity as a post-trauma consequence, however, can affect the victim’s compliance and can be reported as an important symptom and therefore properly mentioned.

The lack of data related to granting or refusal of international protection following the examined forensic assessments does not allow any useful collection of information to evaluate the relationship between the forensic judgements and the local Committee’s final decisions (asylum, subsidiary protection, or refusal).

## Conclusion

The forensic practice in the assessment of the acts of torture claimed by asylum seekers is of utmost juridical importance. The available scientific literature, and especially the literature related to the analysis of the injuries, is scarce and inhomogeneous, and mainly based upon case reports or series. The aim of our study is the highlighting of the recurrence of patterns of injuries in the asylum seekers who claim to be victim of acts of torture and the result of the medicolegal compatibility judgements according to the Istanbul Protocol.

The main findings of the present study are as follows:

- More than 75% of the assessed asylum seekers are over 21 males coming from African countries. About 85% of the migrants claim to have been affected by violent acts in the home country being thus eligible for the asylum grant. Only 15% of cases were ascribed to tortures committed in the countries of transit, mainly in Libya, but no protections are granted by law to these subjects.
- The criteria and procedures provided in the IP result only partly applied in the examined sample that shows an incomplete gradation of the concordance judgements. Only 60.7% of cases are judged “compatible” or “incompatible,” while 39.2% of reports are “inconclusive.”
- In 42.2% of cases, especially in case of deprivation techniques and forced labor of detainees, the physicians did not find any physical signs of torture. About half of the scars—more often those inflicted with generic blunt instruments—are described as nonspecific. The forensic physicians recognize only few injuries as specific even among those which show the highest grade of specificity (whipping, cigarette burn, defense lesions).

The present study highlights also the need for dedicated education and training of the physicians involved in the investigation of cases of alleged tortures, in order to improve the understanding and application of the international guidelines (IP), to better support the activity of the authorities in charge with granting or refusing asylum or protection and to lower the risks of underestimation.

Our sample did not consider the psychological effects of tortures or ill-treatments and the correlation between them and the conclusions of the forensic reports and the Torture Committee decisions. Further studies are necessary to deepen the knowledge in this matter and to study the utility of diagnostic, instrumental, and specialistic tools in the forensic assessments.
